# Mode of treatments and achievement of treatment targets among type 2 diabetes patients with different comorbidities – a register-based retrospective cohort study in Finland

**DOI:** 10.1186/s12875-022-01889-3

**Published:** 2022-11-09

**Authors:** Nazma Akter Nazu, Katja Wikström, Marja-Leena Lamidi, Jaana Lindström, Hilkka Tirkkonen, Päivi Rautiainen, Tiina Laatikainen

**Affiliations:** 1 Department of Public Health, University of Helsinki,, PO BOX 63, 00014 Helsinki, Finland; 2 Institute of Public Health and Clinical Nutrition, University of Eastern Finland, PO BOX 1627, 70211 Kuopio, Finland; 3 Department of Public Health and Social welfare, Finnish Institute for Health and Welfare, PO BOX 30, 00271 Helsinki, Finland; 4 Joint municipal authority for North Karelia Social and Health Services (Siun sote), Tikkamäentie 16, 70210 Joensuu, Finland

**Keywords:** Type 2 diabetes, Mental disorder, Cardiovascular disease, Medication, Treatment targets, HbA1c and LDL

## Abstract

**Aims:**

Type 2 diabetes (T2D) is a progressive disease often associated with comorbidities that complicate the management of T2D and affect the achievement of treatment targets. However, adherence to guidelines and individualized treatments can potentially improve treatment outcomes. This study assessed the association between different glucose lowering and lipid lowering medication lines and the achievement of treatment targets with different comorbidities among a T2D cohort in North Karelia, Finland (2011-12 to 2015-16).

**Methods:**

The data on all diagnosed T2D patients (n = 10,190) in North Karelia were collated retrospectively from regional electronic health records (EHRs). Analyses were performed considering the age, sex, and comorbidities such as cardiovascular diseases (CVD) and any mental disorders (AMD). We analyzed the trends in using glucose lowering and lipid lowering medications and the effect of changes in medication on the achievement of treatment targets among different patient groups.

**Results:**

Metformin was the most common treatment in all patient groups. The use of only metformin declined and the use of metformin and/or other non-insulin medications increased during the follow-up. A Combination of insulin and non-insulin medication was mostly used by T2D patients with both cardiovascular diseases and mental disorders (T2D + CVD + AMD), and the use of insulin increased among this group in follow-up. Achievement of the glucose treatment target deteriorated even after the intensification of medication among all patient groups during the follow-up. A considerably higher number of patients with T2D + AMD and T2D + CVD + AMD did not use lipid lowering medication when compared to the T2D + CVD patients both at baseline and follow-up. However, the achievement of the LDL treatment target improved during the follow-up.

**Conclusion:**

Achievement of the glucose target deteriorated even after the intensification of treatment, and especially among patients with multiple diseases. Many T2D patients with AMD and CVD remained without lipid lowering medication, which needs further attention.

**Supplementary Information:**

The online version contains supplementary material available at 10.1186/s12875-022-01889-3.

## Introduction

Type 2 diabetes (T2D) is one of the fastest growing public health issues and a matter of significant concern for the health care systems globally. The presence of comorbidities or complications alongside T2D further increases the need for health care services and complicates the selection of the appropriate medication and the achievement of treatment targets [1, 2]. Of the various comorbidities, cardiovascular diseases (CVD) are most commonly associated with T2D. A systematic literature review by Einarson et al. reported that almost 32% of all individuals with T2D had CVD and that CVD is a major contributor to T2D mortality [1, 3]. Other comorbid diseases that are frequently found to be associated with T2D and affect the self-management of T2D patients are mental disorders. T2D patients with mental disorders are often reported to have poorer quality diabetes care, more complications, and lower success rate in achieving treatment targets due to their lack of medication adherence and diminished capacity for self-management [4–8].

The principal objective in the management of T2D is to improve the glycemic and lipid control, increase the quality of life of the T2D patient and prevent or delay the onset of complications. It is recommended that T2D management with lifestyle modifications be started on the basis of baseline HbA1c levels and patient profiles [2, 9–11]. Evidence suggests that lifestyle modification is effective in lowering glucose levels, improves lipid levels, and reduces the chances of having diabetes-related complications [12]. However, it is difficult for T2D patients to maintain the required glucose level long-term. This necessitates the introduction of additional glucose lowering medications. Achievement of glucose treatment target becomes challenging with the progression of T2D [13–16], and suboptimum achievement of treatment target has often been reported [17, 18]. A large prospective study conducted in the United Kingdom (UKPDS) showed that approximately half of subjects treated with monotherapy did not achieve the recommended HbA1c target (HbA1c < 7% or 53 mmol/mol) by the end of the three-year follow-up and only 25% had achieved the treatment target by the nine-year follow-up [19]. In their study, Baily et al. suggested that introducing combination therapy in advance may help patients to achieve glycemic targets [20]. Moreover, Buysman et al. found that timely intensification of glucose lowering treatment improves the HbA1c levels of T2D patients [21]. In addition, management of LDL levels is important for T2D patients to avoid major CVD events. The importance of lowering LDL levels is well established, and it is recommended to start statin therapy among T2D patients regardless of the baseline LDL cholesterol level and any prior known CVD events [22]. A systematic review and meta-analysis of 52 randomized trials found that the risk reduction for major cardiovascular events achieved by lowering LDL level by 1 mmol/L is not dependent on baseline LDL level or having T2D or chronic kidney disease [23].

Some studies have assessed the association between different treatment options and the achievement of treatment targets among a specific treatment group (e.g., either monotherapy or combination therapy or insulin) or patient group (e.g., only T2D patients or patients with CVD etc.) [19, 24, 25]. However, few studies have coherently examined the association between different glucose lowering and lipid lowering medication lines with the achievement of treatment targets among different patient groups. Our study intended to observe the association of different glucose lowering and lipid lowering medication lines with the achievement of glucose and lipid treatment target among a T2D cohort in North Karelia, Finland (from 2011 to 12 to 2015-16), considering certain co-morbidities such as cardiovascular diseases and mental disorders.

## Methods

### Study design & setting

This is a register-based retrospective cohort study of 10,197 individuals with T2D. Since the beginning of 2011, a common electronic patient database system (Mediatri) has been used by all municipalities in North Karelia, Finland, covering both primary and secondary level care. We used the regional electronic health records (EHRs) data from 2011 to 2016. We collated data on age, sex, place of residence, visits to both primary and specialized care, e-prescriptions for glucose and lipid lowering medications, permanent diagnosis, and key laboratory markers (HbA1c and LDL levels). The ethics approval for conducting this was received from the Ethics Committee of the Northern Savonia Hospital District on 13 November 2012.

Recommended medications (glucose lowering and lipid lowering medications) were categorized to relevant treatment groups based on the data on e-prescriptions. The use of different medications and the impact of changes in medications on the achievement of glucose and lipid lowering treatment target during the follow-up were then analyzed among patients with only type 2 diabetes (T2D), T2D patients with cardiovascular disease (CVD), T2D patient with any mental disorder (AMD), and T2D patients with both CVD and AMD. The impact of age and sex were also taken into consideration.

### Participants

The participants were identified from the EHRs with the permanent diagnosis of T2D (ICD-10 code E11) or based on the recorded diagnosis on a visit to primary or secondary care during the study period (2011-12 to 2015-16). A total of 10,197 individuals were found to have T2D at the end of 2012. At baseline, we included the participants who were aged 20 or over and were alive at the end of 2012 (n = 10,190). We excluded those who died during the study period (n = 1761), which left 8,429 patients available for full follow-up.

Patients who had cardiovascular diseases (ICD-10 code I20-I25, I46, I48, I50, I63-I66 (except I63.6) and G45) or any mental disorders (ICD-10 code F00-F03, F20-F48 & G30) along with T2D were identified using the permanent diagnoses in the EHRs.

### Variables

According to the guidelines, the general treatment goal for T2D patients is to keep the HbA1c levels at 7% or less (≤ 53 mmol/mol), which can be modified on the basis of patient characteristics. In our study, HbA1C level < 7.0% or 53 mmol/mol was considered as the cut-off for the achievement of glucose treatment target. The recommended LDL treatment target for T2D patients is less than 2.5 mmol/l. However, for patients with a history of CVD, stricter LDL controls need to be followed (LDL < 1.8 mmol/l). We compared the achievement of LDL treatment target among different patient groups and used the general LDL treatment target (LDL < 2.5 mmol/l) as the cut of point [2, 9–11].

The use of glucose medications was categorized into five groups based on the recommended intensification of treatment by national and international diabetes guidelines [2, 10, 11, 26]. The first preference for the management of T2D patients after diagnosis is to reduce the glucose levels by diet and physical activity. If the HbA1c target is not achieved, then initiation of pharmacotherapy is recommended. Usually, the first choice of glucose lowering medication is metformin considering the baseline HbA1c level and contraindications. If the HbA1c level is still more than the target level, then a combination of two or more non-insulin glucose lowering medications can be started, in accordance with the needs of the patient. The new ADA-EASD guidelines emphasize the use of SGLT2 inhibitors or GLP1 analogues as a second choice [10, 11]. The next treatment option could be the use of a combination of any non-insulin medication and insulin. Finally, different types of insulin (short and long acting) can be introduced if more effective glucose management is needed. The recommended duration of the changes between intervention is at least 3–6 months [2, 10, 11, 26].

We categorized glucose lowering medication into (1) No medication group (comprising diet and physical activity), (2) Metformin only group (Anatomic Therapeutical Chemical (ATC) code A10BA02), (3) Metformin and/or other non-insulin medication group (medication starting with ATC code A10BA, A10BB, A10BD, A10BG, A10BH, A10BK, A10BX or A10BJ), (4) Combination of insulin and non-insulin medication (all medication with ATC code A10B together with A10AB or A10AC or A10AD or A10AE) and (5) Only insulin therapy group (ATC code A10AB, A10AC, A10AD or A10AE).

Lipid medications were categorized into four groups based on the use of statins and ezetimibe: (1) No therapy group (no statins nor ezetimibe), (2) Low intensity group (ATC code C10AA01–10 mg; C10AA02–20 mg; C10AA03–10-20 mg; C10AA04–20-40 mg; C10AA08–1 mg; C10BX02), (3) Moderate intensity (ATC code C10AA01–20-40 mg; C10AA02–40 mg; C10AA03–40-80 mg; C10AA04–80 mg; C10AA05–10-20 mg; C10AA07–5-10 mg; C10BA01, C10BA03, C10BA04) and (4) High intensity lipid lowering medication group (C10AA05–40-80 mg; C10AA07–20-40 mg; a readily available combination of statin and ezetimibe – C10BA02; or any statin (ATC code starting with C10AA) along with ezetimibe, ATC code C10AX09).

We calculated the changes in glucose lowering and lipid lowering treatment using the ordinal number of categories as follows; (Use of glucose lowering/lipid lowering medication in 2015-16 – Use of glucose lowering/lipid lowering medication in 2011-12). The positive (+) values reflect intensification of treatment, 0 is no change, and negative (-) values reflect de-intensification of treatment. For example, regarding glucose lowering medication, if the patient was in the Metformin group (category 2) in 2015-16 and in no medication group (category 1) in 2011-12, then the change in treatment was interpreted as (2 − 1 = 1) intensified. We then analyzed the impact of changes in treatment on the achievement of glucose and lipid lowering treatment target by different patient groups.

Considering the comorbid condition present along with the T2D, the patient groups were divided into 4 categories. (1) Only T2D (ICD-10 code E11), (2) T2D + CVD (ICD-10 code E11 + ICD-10 code I20-I25, I46, I48, I50, I63-I66 (except I63.6) and G45), (3) T2D + AMD (ICD-10 code E11 + ICD-10 code F00-F03, F20-F48 & G30), (4) T2D + CVD + AMD (ICD-10 code E11 + ICD-10 code I20-I25, I46, I48, I50, I63-I66 (except I63.6) and G45) + ICD-10 code F00-F03, F20-F48 & G30)

### Biochemical methods

HbA1c and LDL samples were analyzed by the turbidimetric inhibition immunoanalysis method (TINIA) and photometric direct enzymatic method, respectively, in the Eastern Finland laboratory (ISLAB). The results were standardized to International Federation of Clinical Chemistry (IFCC) units.

### Statistical method

The basic patient characteristics were described with counts, percentages and mean values. Use of medications (Glucose lowering and Lipid lowering medications) and the achievement of treatment targets (HbA1c < 7% or 53 mmol/mol and LDL < 2.5 mmol/l) by different patient groups were expressed with percentages. To assess the differences in the use of medication and changes in the treatment during follow-up by different patient groups, logistic regression models with generalized estimating equations (GEE) were used, as repeated measurements can be accommodated with this model. Along with unadjusted results, age and sex-adjusted results were also presented. P-values of less than 0.05 were regarded as statistically significant. We have used transition plots to illustrate the change trends in the use of glucose lowering medications by the different patient groups during the follow-up.

## Results

Table 1 represents the basic patient characteristics. The mean age of the patients was 66 years and 47% of them were women. Among the patient groups whose HbA1c or LDL was measured both in 2011-12 and 2015-16, the mean HbA1c level was the highest among T2D + CVD and T2D + CVD + AMD patients, and the highest mean LDL levels were observed among Only T2D and T2D + AMD patients at the baseline (2011-12). Metformin was the most used glucose lowering medication both in 2011-12 and 2015-16. The mean age of the patient’s using metformin was 65 years and 52% of them were women. The mean HbA1c level worsened among those whose medication was changed and those who remained in the same medication category, at both baseline and follow-up. Regarding the lipid lowering medications, moderate intensity lipid lowering medications were more common among men, and the mean age was 66 years. The mean LDL levels improved among those who were in moderate or high intensity lipid lowering medication therapy and remained unchanged in those who were in no therapy or low intensity lipid lowering medication therapy, at both baseline and follow-up. In addition, the mean LDL level improved among patients whose medication changed during the follow-up (Table 1).

**Table 1:** Basic characteristics of the patients

Categories	N (%)		Mean age in years (SD)	Sex Women (%)	Mean HbA1c ^f^ (%)		Mean LDL ^g^ (mmol/l)	
					2011-12 (SD)	2015-16 (SD)	2011-12 (SD)	2015-16 (SD)
Patient groups ^a^								
T2D only	4440	(53)	64 (10.9)	48	6.5 (1.11)	6.8 (1.22)	2.6 (0.81)	2.5 (0.85)
T2D + CVD	2544	(30)	71 (9.41)	39	6.7 (1.14)	7.0 (1.25)	2.3 (0.77)	2.1 (0.82)
T2D + AMD	942	(11)	60 (12.6)	61	6.6 (1.31)	6.8 (1.35)	2.6 (0.84)	2.5 (0.93)
T2D + CVD + AMD	503	(6)	70 (11.7)	55	6.7 (1.26)	7.1 (1.41)	2.4 (0.87)	2.3 (0.87)
Glucose lowering medication ^b^								
No medication	627	(7)	68 (12.6)	52	5.7 (0.35)	5.8 (0.37)	2.8 (0.89)	2.6 (1.00)
Metformin only	2009	(24)	65 (10.9)	52	5.8 (0.38)	6.1 (0.53)	2.5 (0.81)	2.4 (0.84)
Metformin and/or other non-insulin medication	1317	(16)	66 (10.5)	46	6.3 (0.67)	6.6 (0.85)	2.5 (0.77)	2.4 (0.82)
Combination of insulin and non-insulin medication	1810	(21)	65 (11.0)	42	7.5 (1.30)	7.7 (1.33)	2.4 (0.79)	2.2 (0.82)
Only insulin therapy	329	(4)	69 (12.0)	43	7.5 (1.43)	7.7 (1.48)	2.5 (0.86)	2.3 (0.96)
Medication changed ^c^	2337	(28)	66 (12.0)	46	6.5 (1.03)	6.9 (1.19)	2.6 (0.84)	2.4 (0 89)
Lipid lowering medication ^d^								
No therapy	1869	(22)	64 (13.7)	51	6.5 (1.17)	6.8 (1.22)	2.8 (0.71)	2.8 (0.74)
Low intensity	467	(6)	71 (10.4)	60	6.5 (0.98)	6.9 (1.29)	2.2 (0.63)	2.2 (0.64)
Moderate intensity	3740	(44)	66 (10.5)	45	6.6 (1.11)	6.9 (1.20)	2.3 (0.77)	2.2 (0.80)
High intensity	633	(8)	65 (9.45)	33	6.7 (1.20)	7.0 (1.32)	2.4 (0.82)	2.2 (0.91)
Medication changed ^e^	1720	(20)	66 (10.9)	47	6.6 (1.27)	7.0 (1.29)	2.8 (0.86)	2.5 (0.99)

Selection criteria in different categories: All patients (a-g) were aged ≥ 20 and were alive by the end of 2016, (b) patients who were in the same glucose lowering medication group both in 2011-12 and 2015-16, (c) whose glucose lowering medication was changed during the follow-up, (d) patients who were in the same lipid lowering medication group both in 2011-12 and 2015-16, (e) whose lipid lowering medication was changed during the follow-up, (f) whose HbA1c measured both in 2011-12 and 2015-16, (g) whose LDL measured both in 2011-12 and 2015-16. SD = Standard deviation, CVD = cardiovascular disease (I20-I25, I46, I48, I50, I63-I66 (except I63.6) and G45), AMD = any mental disorder (ICD-10 code F00-F03, F20-F48 & G30)

**Fig. 1:**
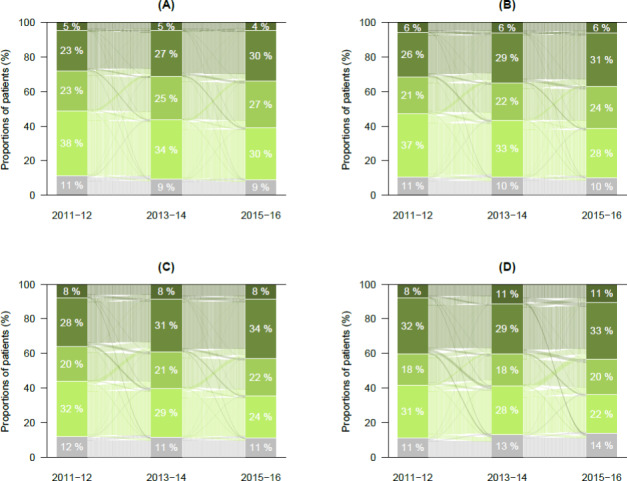
Transition of patients within glucose lowering medication group by patient category. (A) only T2D, (B) T2D & Any Mental Disorder (AMD), (C) T2D & Cardiovascular disease (CVD) and (D) T2D & CVD & AMD. Colour gradients indicate the medication categories; from bottom grey colour to upward categories are No medication, Metformin only, Metformin and/or other non-insulin medication, Combination of insulin and non-insulin medication, and Only Insulin therapy groups, respectively

The use of glucose lowering medications by different patient groups are presented in Table 2. The proportion of patients using metformin alone declined statistically significantly during the follow-up among all patient groups. Metformin and/or other non-insulin medications were mostly used by patients with only T2D, at both baseline and follow-up, and the use increased statistically significantly during the follow-up among all patient groups except among those with T2D + CVD + AMD. A Combination of insulin and non-insulin medication seems to be the most common choice of treatment for those with T2D + CVD + AMD. Furthermore, there were no statistically significant changes in the use of only insulin therapy during the follow-up among all patient groups, except those with T2D + CVD + AMD, who showed an increasing trend in the use of only insulin therapy (Table 2). A transition plot was drawn up in order to observe the movement of patients between different glucose lowering medication groups. We found there was very little transition of patients from one medication category to another. Moreover, these transitions were similar for all patient groups (Fig. 1).

**Table 2:** Proportion of use of glucose lowering medication by different patient group

	only T2D (n = 4440)			T2D + CVD (n = 2544)			T2D + AMD (n = 942)			T2D + CVD + AMD (n = 503)		
	2011-12	2015-16	P-value	2011-12	2015-16	P-value	2011-12	2015-16	P-value	2011-12	2015-16	P-value
Medication group:												
No medication	11	9	< 0.001	12	11	0.098	11	10	0.722	11	14	0.047
Metformin only	38	30	< 0.001	32	24	< 0.001	37	28	< 0.001	31	23	< 0.001
Metformin and/or other non-insulin medication	23	27	< 0.001	20	22	0.032	21	24	0.018	18	21	0.106
Combination of insulin and non-insulin medication	23	30	< 0.001	28	34	< 0.001	26	31	< 0.001	32	33	0.083
Only Insulin therapy	5	4	0.241	8	9	0.306	6	6	0.758	8	11	0.042

P-value from the logistic regression models with generalized estimating equations (GEE) for the changes in the use of glucose lowering medications from baseline (2011-12) to follow-up (2015-16) by different patient groups. Adjustment for age and sex did not change the P-value. CVD = cardiovascular disease (I20-I25, I46, I48, I50, I63-I66 (except I63.6) and G45), AMD = any mental disorder (ICD-10 code F00-F03, F20-F48 & G30)

Table 3 illustrates the use of lipid lowering medications among different patient groups. A considerably higher number of patients with T2D + AMD and T2D + CVD + AMD were not using medication both at baseline and follow-up, when compared with the T2D + CVD patients. Moderate intensity lipid lowering medications were the mostly used medication in all disease groups, especially among those with T2D + CVD both at baseline and follow-up (57% and 56%, respectively). A statistically significant increase in the use of high intensity lipid lowering medication was observed among those with T2D + CVD (16% and 22% in 2011-12 and 2015-16, respectively) compared with those with T2D + CVD + AMD (14% and 16% in 2011-12 and 2015-16, respectively) during the follow-up (Table 3).

**Table 3:** Proportion of use of lipid lowering medication by different patient group

	only T2D (n = 4440)			T2D + CVD (n = 2544)			T2D + AMD (n = 942)			T2D + CVD + AMD (n = 503)		
	2011-12	2015-16	P-value	2011-12	2015-16	P-value	2011-12	2015-16	P-value	2011-12	2015-16	P-value
Medication group:												
No therapy	37	30	< 0.001	18	14	< 0.001	40	36	< 0.001	31	29	0.204
Low intensity	8	8	0.004	9	7	0.003	8	7	0.354	8	5	0.022
Moderate intensity	50	54	< 0.001	57	56	0.178	49	52	0.048	48	50	0.215
High intensity	5	9	< 0.001	16	22	< 0.001	3	5	< 0.001	14	16	0.058

P-value from the logistic regression models with generalized estimating equations (GEE) for the changes in the use of lipid lowering medications from baseline (2011-12) to follow-up (2015-16) by different patient groups. Adjustment with age and sex did not change the P-value. CVD = cardiovascular disease (I20-I25, I46, I48, I50, I63-I66 (except I63.6) and G45), AMD = any mental disorder (ICD-10 code F00-F03, F20-F48 & G30)

Changes in the use of glucose lowering medication during the follow-up and its association with the achievement of treatment target (HbA1c < 7% or 53 mmol/mol) are described in Table 4. The achievement of treatment target deteriorated even after the intensification of medication among all patient groups during the follow-up. The changes in the achievement of treatment targets between the different patient groups were not statistically significantly different even after adjustment for age and sex, and they were irrespective to changes in medication intensity (Table 4). The deterioration in achievement of treatment targets was observed also when using higher cut-off point for HbA1c ≤ 8% (64 mmol/l) (Supplementary Table 1). The prevalence of those who met the treatment targets obviously increased.

**Table 4:** Achievement of treatment target (HbA1c < 7% or 53 mmol/mol) by the changes in use of glucose lowering medication

	Changes in the use of glucose lowering medication			Overall achievement of treatment target
	Intensification of medication	No change in medication	De-intensification of medication	
Patient categories: ^a^				
**T2D only**	(n = 530)	(n = 2409)	(n = 111)	(n = 3050)
Proportion of patients undergoing changes in glucose medication, %	17.4	79.0	3.6	
Target met 2011-12, %	83.0	74.0	93.7	76.3
Target met 2015-16, %	63.4	68.1	87.4	68.0
Change in achievement of treatment target (%)	-19.6	-5.9	-6.3	-8.3
**T2D + CVD**	(n = 363)	(n = 1493)	(n = 110)	(n = 1966)
Proportion of patients undergoing changes in glucose medication, %	18.5	75.9	5.6	
Target met 2011-12, %	81.3	67.9	90.0	71.3
Target met 2015-16, %	63.4	60.4	89.1	62.6
Change in achievement of treatment target (%)	-17.9	-7.5	-0.9	-8.7
**T2D + AMD**	(n = 127)	(n = 508)	(n = 40)	(n = 675)
Proportion of patients undergoing changes in glucose medication, %	18.8	75.3	5.9	
Target met 2011-12, %	88.2	68.7	95.0	73.9
Target met 2015-16, %	66.9	61.0	80.0	63.3
Change in achievement of treatment target (%)	-21.3	-7.7	-15.0	-10.6
**T2D + CVD + AMD**	(n = 57)	(n = 290)	(n = 32)	(n = 379)
Proportion of patients undergoing changes in glucose medication, %	15.0	76.5	8.4	
Target met 2011-12, %	78.9	64.5	87.5	68.6
Target met 2015-16, %	56.1	59.0	84.4	60.7
Change in achievement of treatment target (%)	-22.8	-5.5	-3.1	-7.9
P-value ^b^	0.683	0.835	0.157	0.712

(a) Selection criteria of the patients: Age ≥ 20, alive by the end of 2016 and whose HbA1c measured both in 2011-12 and 2015-16 (n = 6070). (b) P-value for the differences in the changes in use of glucose lowering medication during the follow-up between different patient groups; logistic regression models with GEE. Adjustment for age and sex did not change the P-value. CVD = cardiovascular disease (I20-I25, I46, I48, I50, I63-I66 (except I63.6) and G45), AMD = any mental disorder (ICD-10 code F00-F03, F20-F48 & G30)

Table 5 presents the changes in the use of lipid lowering medication and its association with the achievement of treatment target (LDL < 2.5 mmol/l). The improvement in the achievement of lipid treatment target was observed during the follow-up in all patient groups among those whose medication was intensified. The biggest improvement was observed among those with T2D + AMD. In contrast, the de-intensification of lipid lowering medication was negatively associated with the achievement of lipid treatment target, with no statistically significant differences between patient groups. (Table 5).

**Table 5:** Achievement of LDL treatment target (LDL < 2.5 mmol/l) by the changes in use of lipid lowering medication

	Changes in the use of lipid lowering medication			Overall achievement of treatment target
	Intensification of medication	No change in medication	De-intensification of medication	
Patient categories ^a^				
**T2D only**	(n = 500)	(n = 2264)	(n = 137)	(n = 2901)
Proportion of patients undergoing changes in lipid medication, %	17.2	78.0	4.7	
Target met 2011-12, %	30.4	52.7	30.7	47.8
Target met 2015-16, %	62.0	57.4	19.7	56.4
Change in achievement of treatment target (%)	31.6	4.7	-11.0	8.6
**T2D + CVD**	(n = 295)	(n = 1458)	(n = 92)	(n = 1845)
Proportion of patients undergoing changes in lipid medication, %	16.0	79.0	5.0	
Target met 2011-12, %	49.5	70.1	48.9	65.7
Target met 2015-16, %	71.9	73.7	39.1	71.7
Change in achievement of treatment target (%)	22.4	3.6	-9.8	6
**T2D + AMD**	(n = 86)	(n = 495)	(n = 27)	(n = 608)
Proportion of patients undergoing changes in lipid medication, %	14.1	81.4	4.4	
Target met 2011-12, %	16.3	53.3	44.4	47.7
Target met 2015-16, %	57.0	58.2	14.8	56.1
Change in achievement of treatment target (%)	40.7	4.9	-29.6	8.4
**T2D + CVD + AMD**	(n = 46)	(n = 248)	(n = 16)	(n = 310)
Proportion of patients undergoing changes in lipid medication, %	14.8	80.0	5.2	
Target met 2011-12, %	34.8	67.7	43.8	61.6
Target met 2015-16, %	67.4	70.6	18.8	67.4
Change in achievement of treatment target (%)	32.6	2.9	-25.0	5.8
P-value ^b^	0.043	0.983	0.318	0.731

(a) Patient selection criteria: Age ≥ 20, alive by the end of 2016 and whose LDL measured both in 2011-12 and 2015-16 (n = 5664). (b) P-value for the differences in the changes in use of lipid lowering medication during the follow-up between different patient groups; logistic regression models with GEE. Adjustment for age and sex did not change the P-value. CVD = cardiovascular disease (I20-I25, I46, I48, I50, I63-I66 (except I63.6) and G45), AMD = any mental disorder (ICD-10 code F00-F03, F20-F48 & G30)

## Discussion

This study describes the use of glucose lowering and lipid lowering medication and their association with the achievement of treatment targets (HbA1c < 7% or 53 mmol/mol and LDL < 2.5 mmol/l) among T2D patients with and without comorbidities in Finland during a six-year follow-up. “Metformin only” was the most common mode of treatment in all patient groups. However, the use of metformin only declined and the use of metformin and/or other non-insulin medications increased during the follow-up. A Combination of insulin and non-insulin medication was mostly used for the T2D patients who had both mental disorders and CVD. Moreover, the use of only insulin also increased among this group during the follow-up. Despite the intensification of medication, the achievement of glucose treatment target deteriorated among all patient groups during the follow-up. The achievement of LDL treatment target improved during the follow-up. However, many patients with T2D + AMD and T2D + CVD + AMD were not using any lipid lowering medication, at both baseline and follow-up.

The evidence demonstrates that it is difficult for the T2D patient to maintain a steady glucose level in the long-term because of the gradual deterioration of β cells [27]. In addition, the guidelines suggest that pharmacological treatment should be initiated without delay if the glucose target is not met through behavioral changes [2, 9]. We observed that the proportion of patients without glucose lowering medication declined among the T2D only patient group during the follow-up. However, an increasing trend in not using any medication was observed among those who had both CVD and AMD along with T2D. A possible reason for this finding might be the fact that this particular patient group (T2D + CVD + AMD) comprised older adults, and the evidence shows that older adult patients lack motivation to use medication, they often forget to take medicine, they get confused when multiple medications are prescribed, and they often fear side effects, which makes them avoid taking their medication [28, 29].

In our study, metformin was the mostly used medication at baseline among all patient groups. This corresponds with the treatment protocol of different national and international guidelines, as metformin is considered the first-line pharmacological treatment for T2D patients [2, 10, 11, 26]. Prior studies have shown that metformin improves the glycemic control of T2D patients, rarely causes hypoglycemia, and is remarkably safe [30, 31]. However, we found that the use of metformin only declined during the follow-up. Long-term use of metformin may initiate vitamin B12 deficiencies, gastro-intestinal side effect, and renal impairment [2, 32], which might partly explain the reduction in the use of metformin during the follow-up. We noticed that the use of metformin and/or other non-insulin medication increased among all patient groups during the follow-up. This is a good indication, as the literature suggests that early introduction of a combination therapy gives better, and longer-term benefits compared with monotherapy [33]. Monotherapy has been found to not be sufficient to maintain the glucose levels for most T2D patients because of the progressive nature of the disease [34]. Moreover, the introduction of an additional glucose lowering agent is recommended if monotherapy fails to achieve or maintain the glycemic target [2, 26]. It has previously recommended to treat only the patients with a known history of CVD, with GLP-1 and SGLT2 inhibitors. However, newer guidelines suggest starting GLP-1 and SGLT2 inhibitors as a secondary medication choice for those who are even at risk of CVD [10, 11]. A Combination of insulin and non-insulin medication was the most common treatment choice for those with T2D + CVD + AMD, at both baseline and follow-up. This is most likely due to the older age and higher HbA1c level at baseline in this particular patient group.

We noticed some changes in the use of glucose lowering medication during the follow-up. Surprisingly, despite the intensification of glucose lowering medication, achievement of the glucose treatment target (HbA1c < 7% or 53 mmol/mol) declined during the follow-up among all patient groups, and especially among those with multiple comorbidities (T2D + CVD + AMD). Our finding is in line with Harris et al., Piette et al., and the UKPDS33 study. They also found an association between multiple co-morbidity and suboptimum achievement of glycemic treatment target [4, 24, 35]. Poor adherence to the complex treatment regimen for multiple diseases and progression of disease seems to be the underlying reason for the poor achievement of treatment targets [4, 15, 16, 35]. Besides, harm of intensive glucose control shown by big trials may have influenced the treatment strategies for patients with multiple comorbidities [36, 37]. Relaxed treatment target for the older and multiple comorbid patient group by the guidelines may also cause poor achievement of treatment targets [2, 9–11]. In addition, mental disorders and T2D have been found to have a bi-directional relation. Antipsychotic drugs have a negative effect on glycemic control. A study in USA analyzing the effects of common antipsychotic medications on glucose and lipid levels showed that antipsychotics are associated with weight gain, elevated glucose and triglyceride levels [38, 39]. Additionally, strict glucose control is not recommended for CVD patients because of the risk of hypoglycemia [9]. It is well established that early intensification of medication helps to achieve the treatment targets sooner, delays disease progression, and reduces the chances of development of complications [20]. In our study, the transition plot of changes in the use of glucose lowering medications by different patient groups shows very little movement of patients from one medication group to another. This indicates that the medication may not be intensified enough during the follow-up, and it may have affected the achievement of treatment targets. Thus, it is unclear whether the intensification of medication and the time of introducing intensification was appropriate for the different patient groups.

Interestingly, our study found that a considerably larger number of patients with T2D + CVD + AMD were in the no lipid medication group during the follow-up compared with those having T2D + CVD. It is especially alarming that patients with CVD are not using statins. Dyslipidemia and T2D are considered as crucial risk factors for myocardial infarction, and it is recommended that lipid lowering medication should be started in T2D patients even without a CVD diagnosis [40, 41]. Thus, our finding indicates an evidence-treatment gap, showing that patients with mental disorders are not well managed despite having CVD. This finding is in line with earlier literature which also shows suboptimum management of patients with mental disorder and CVD [42–44]. There is need of immediate action to avoid severe cardiovascular outcomes among this patient group.

We observed an improvement in the achievement of the lipid target during the follow-up in all patient groups among those whose treatment was intensified. Our finding is in line with Gant et al., who investigated the achievement of LDL treatment target among T2D patients and reported that increasing statin treatment has the potential to improve the achievement of LDL treatment target [45]. In our study, achievement of the LDL treatment target was the highest among patients with T2D + AMD and T2D + CVD + AMD whose treatment was intensified. This further emphasizes the importance of introducing lipid lowering medications for those with T2D + AMD and T2D + CVD + AMD, who were not receiving any lipid lowering medications during the follow-up. Another interesting finding was that, despite the improvement in the achievement of LDL treatment target among T2D patients with only CVD and CVD along with AMD, still only less than 75% achieved the LDL target of < 2.5 mmol/l during the follow-up. It should be remembered that the real target for any CVD patient should be LDL < 1.8 mmol/l [2, 11]. This signifies that there is still scope for improvement and that T2D patients with CVD, AMD or both need further attention.

This study has several strengths. One of the most important strengths is the large and comprehensive sample. Regional EHRs enabled us to collate the data on all diagnosed T2D patients in North Karelia, thus also avoiding selection bias. This study provides follow-up information, spanning a period of six years, on the pharmacological management of T2D among different patient groups in a real-life setting. This helps us visualize the possible opportunities to improve the T2D treatment strategies for the T2D cohort of North Karelia. As all laboratory test results and e-prescriptions are recorded directly in the EHRs, any missing information, incorrect HbA1c and LDL values, or wrong medication information were avoided. In addition, the same standardized methods for testing HbA1c levels are used by all municipalities in North Karelia, which ensures the comparability of the data. This study has some limitations as well. One such is that only the information on prescriptions was available from the EHRs. Consequently, we cannot be certain if the patient collected the medication and took it properly. This might influence the achievement of treatment target. The second limitation was the lack of information regarding disease duration. Although we have included information on T2D patients from both primary and secondary level care, there might be some patients who did not use the service regularly or who also used private health care services during the follow-up period. In addition, we were not able to retrieve information about hypertension and BMI from the EHRs so that they could be taken into account for the analysis although they contain very valuable information for the management of T2D. However, the contents of the EHRs are updating all the time and expected to provide valuable information in near future.

## Conclusion

In general, it seemed that the management guidelines are followed quite evenly among the different patient groups. However, despite the intensification of treatment, the achievement of glucose target deteriorated. This was especially true for those patients with multiple diseases, indicating the challenges in the management of progressive disease among patients with multi-morbidities. This study suggests a need for more focused and individualized treatment processes for these patients. Although the achievement of LDL treatment target improved, showing a clear association with the intensification of lipid-lowering treatment, many patients with AMD and CVD remained in the no medication group. Accordingly, T2D patients with co-morbid conditions need further attention in order to prevent macrovascular complications.

## Electronic supplementary material

Below is the link to the electronic supplementary material.

Supplementary Material 1

## Data Availability

The health records data analyzed in the current study is confidential, and according to the Finnish Personal data act, cannot be made publicly available to protect the privacy of patients. Data are available from the corresponding author on reasonable request with the permission from the joint municipal authority for North Karelia social and health services.
