# Supplementary material for: Mode of treatments and achievement of treatment targets among type 2 diabetes patients with different comorbidities – a register-based retrospective cohort study in Finland
Source: BMC Prim Care. 2022 Nov 9;23:278. doi: 10.1186/s12875-022-01889-3 (PMC9644526; doi:10.1186/s12875-022-01889-3)
Supplement: Supplementary file 1 — Supplementary Material 1 [file 12875_2022_1889_MOESM1_ESM.docx]

Supplement Table 1: Achievement of treatment target (HbA1c ≤ 8% or 64 mmol/mol) by the changes in use of glucose lowering medication.

|  | Changes in the use of glucose lowering medication | | | Overall achievement of treatment target |
| --- | --- | --- | --- | --- |
|  | Intensification of medication | No change in medication | De-intensification of medication |  |
| Patient categories: ^a^ |  |  |  |  |
| **T2D only** | (n = 530) | (n = 2409) | (n = 111) | (n = 3050) |
| Proportion of patients undergoing changes in glucose medication, % | 17.4 | 79.0 | 3.6 |  |
| Target met 2011-12, % | 96.2 | 88.5 | 99.1 | 90.2 |
| Target met 2015-16, % | 85.1 | 84.1 | 96.4 | 84.7 |
| Change in achievement of treatment target (%) | -11.1 | -4.4 | -2.7 | -5.5 |
| **T2D + CVD** | (n = 363) | (n = 1493) | (n = 110) | (n = 1966) |
| Proportion of patients undergoing changes in glucose medication, % | 18.5 | 75.9 | 5.6 |  |
| Target met 2011-12, % | 97.0 | 86.7 | 99.1 | 89.3 |
| Target met 2015-16, % | 84.6 | 79.2 | 97.3 | 81.2 |
| Change in achievement of treatment target (%) | -12.4 | -7.5 | -1.8 | -8.1 |
| **T2D + AMD** | (n = 127) | (n = 508) | (n = 40) | (n = 675) |
| Proportion of patients undergoing changes in glucose medication, % | 18.8 | 75.3 | 5.9 |  |
| Target met 2011-12, % | 96.1 | 84.1 | 100 | 87.3 |
| Target met 2015-16, % | 81.9 | 80.3 | 97.5 | 81.6 |
| Change in achievement of treatment target (%) | -14.2 | -3.8 | -2.5 | -5.7 |
| **T2D + CVD + AMD** | (n = 57) | (n = 290) | (n = 32) | (n = 379) |
| Proportion of patients undergoing changes in glucose medication, % | 15.0 | 76.5 | 8.4 |  |
| Target met 2011-12, % | 96.5 | 81.4 | 93.8 | 84.7 |
| Target met 2015-16, % | 73.7 | 76.6 | 90.6 | 77.3 |
| Change in achievement of treatment target (%) | -22.8 | -4.8 | -3.2 | -7.4 |
| P-value ^b^ | 0.774 | 0.152 | 0.727 | 0.771 |
| (a) Selection criteria of the patients: Age ≥20. alive by the end of 2016 and whose HbA1c measured both in 2011-12 and 2015-16 (n = 6070)  (b) P-value for the differences in the changes in use of glucose lowering medication during the follow-up between different patient groups; logistic regression models with GEE. Adjustment for age and sex did not change the P-value.  CVD = cardiovascular disease (I20-I25, I46, I48, I50, I63-I66 (except I63.6) and G45), AMD = any mental disorder (ICD-10 code F00-F03, F20-F48 & G30). | | | | |
